# Review of Professor Peirce’s Paper, “Pyorrhœa Alveolaris”

**Published:** 1894-04

**Authors:** S. H. Guilford

**Affiliations:** Philadelphia


### REVIEW OF PROFESSOR PEIRCE'S PAPER, "PYORRHŒA ALVEOLARIS."[1]

BY S. H. GUILFORD, D.D.S., PHILADELPHIA.

In undertaking to express my views upon the subject under consideration, I regret that I have neither any new views to present nor new methods of treatment to offer.

In common with other busy practitioners, it has been my lot to encounter more cases of this disease than I cared for, and to meet with less success in its treatment than I had hoped to attain. The insidious character of the disease and its seemingly growing prevalence certainly lay upon us, as professional men, an increasing obligation to try to fathom the mystery of its etiology, in order that our remedial measures may have a scientific foundation.

The first one, so far as I know, to give this disease anything like the attention it deserved was Dr. Riggs, who, while he paid little attention to its etiology, deserves great credit for the assiduity with which he labored for the amelioration of the condition and the success which attended his efforts.

---

[1] Read before the Eighth District Dental Society, Buffalo, N. Y., February 26, 1894.

His treatment was largely mechanical, but it was a long step in the right direction, for it not only afforded relief from suffering, but measurably checked the progress of the disease. The removal by instrumentation of the only apparent cause of the trouble was the best treatment that had been suggested, but it fell short of real efficiency, because the remote cause of the trouble had not yet been discovered, and all treatment was therefore palliative rather than remedial or preventive. We were aware of the calcareous nature of the deposit on the roots of the teeth, which was the immediate cause of the disease, and also recognized its general similarity to the calcic deposits found upon the crowns of teeth, but it was left for Ingersoll or Black (one or both) to determine that it was a deposit from the blood, and that any means adopted for its prevention must deal with causes of a constitutional character.

Following up this line of investigation, Professor Peirce has lately advanced the theory, based upon analyses of the deposit, that the primary cause of the disease is a uric-acid diathesis. His analyses showed the presence of the urates of calcium and sodium, in addition to the phosphate and carbonate of lime, which make up the bulk of all calcic deposits.

As these salts are similar to those found in the exudations of gouty subjects, he very naturally concludes that the origin of the two diseases, gout and pyorrhœa alveolaris, is identical, and in each case is due to an excess of uric acid in the blood-plasma.

Now, while there is a degree of plausibility in the theory as set forth in the INTERNATIONAL DENTAL JOURNAL for January of this year, it lacks confirmatory evidence in several particulars.

In the first analysis given, only three specimens had been examined, and of these but two showed the presence of uric acid in any appreciable quantity. In the second analysis, made by Professor Brubaker, "some six or eight specimens were examined, in three of which only urate of soda crystals were found." It will thus be seen that at most but eleven specimens had been examined, and of these only five, or less than one-half, gave positive evidence of the presence of urates.

Certainly, the number of analyses was entirely too small upon which to base a theory, and their results too unfavorable to serve as confirmatory proof. In describing the difference between serumal and salivary calculus, he speaks of the former as being *constitutional* and the latter *local* in its origin. Now, to my mind, if one is constitutional, the other is also, for the saliva must receive its calcic constituents from the blood, since there is no other source from which they can be derived. Both forms of calcic deposits being therefore derived from the blood, the one directly, and the other indirectly through the medium of the saliva, the experiments would have been more complete and of greater value had both varieties of the deposit been examined for urates. It is possible that they would have been found in each, and in such event would hardly have been considered confirmatory of the theory advanced.

If it should be granted or proved, however, that the exudations and deposits are the same both in gout and pyorrhœa, and their origin similar, we ought to find the two diseases—or rather the two manifestations of the same disease—commonly associated in the same individual. Is this the case? Although Professor Peirce and certain others think they have noticed this relationship, we seriously doubt whether it exists in many cases.

That both should be found occasionally in the same individual is not to be wondered at, for gouty manifestations are far from uncommon and pyorrhœa is multiplying its victims very rapidly; but two diseases of totally different origin are frequently found in the same individual.

To prove a common origin, they would both have to be amenable to the same line of treatment, and until the identity of the two conditions is thus established, we must class ourselves among the doubters or unbelievers in the theory advanced. In all the cases of pyorrhœa that have come under my observation, I have found very few in which gouty manifestations were certainly present, while the number of individuals suffering with gout unattended by symptoms of pyorrhœa is excessively large. The experience of others in this respect may be different from mine, but having lived to see so many theories based upon a slender foundation first accepted, then investigated, and finally disproved and discarded, I, for one, have come to the point of requiring for the acceptance of any new theory a stronger array of facts and confirmatory evidence than has been presented by Dr. Peirce in support of his hypothesis.

